# Retrospective unbiased plasma lipidomic of progressive multiple sclerosis patients-identifies lipids discriminating those with faster clinical deterioration

**DOI:** 10.1038/s41598-020-72654-8

**Published:** 2020-09-24

**Authors:** Mario Amatruda, Maria Petracca, Maureen Wentling, Benjamin Inbar, Kamilah Castro, Emily Y. Chen, Michael A. Kiebish, Keith Edwards, Matilde Inglese, Patrizia Casaccia

**Affiliations:** 1 Advanced Science Research Center at the Graduate Center of the City University of New York, 85 Saint Nicholas Terrace, 4th Fl, New York, NY 10031 USA; 2 Department of Neurology, Icahn School of Medicine at Mount Sinai, New York, NY USA; 3 Department of Neurosciences, Reproductive and Odontostomatological Sciences, Federico II University, Naples, Italy; 4 Department of Neuroscience, Graduate School of Biomedical Sciences, Icahn School of Medicine at Mount Sinai, New York, NY USA; 5 BERG LLC, Framingham, MA USA; 6 MS Center of Northeastern New York, Latham, NY USA; 7 Department of Radiology, Icahn School of Medicine at Mount Sinai, New York, NY USA; 8 Department of Neurosciences, Rehabilitation, Ophthalmology, Genetics, Maternal and Child Health (DiNOGMI) and Center of Excellence for Biomedical Research (CEBR), Neurologic Clinic, University of Genoa, Genoa, Italy

**Keywords:** Neuroscience, Diseases of the nervous system, Multiple sclerosis

## Abstract

The disease course of patients with a confirmed diagnosis of primary progressive multiple sclerosis (PPMS) is uncertain. In an attempt to identify potential signaling pathways involved in the evolution of the disease, we conducted an exploratory unbiased lipidomic analysis of plasma from non-diseased controls (n = 8) and patients with primary progressive MS (PPMS, n = 19) and either a rapid (PPMS-P, n = 9) or slow (PPMS-NP, n = 10) disease course based on worsening disability and/or MRI-visible appearance of new T2 lesions over a one-year-assessment. Partial least squares-discriminant analysis of the MS/MS^ALL^ lipidomic dataset, identified lipids driving the clustering of the groups. Among these lipids, sphingomyelin-d18:1/14:0 and mono-hexosylceramide-d18:1/20:0 were differentially abundant in the plasma of PPMS patients compared to controls and their levels correlated with MRI signs of disease progression. Lyso-phosphatidic acid-18:2 (LPA-18:2) was the only lipid with significantly lower abundance in PPMS patients with a rapidly deteriorating disease course, and its levels inversely correlated with the severity of the neurological deficit. Decreased levels of LPA-18:2 were detected in patients with more rapid disease progression, regardless of therapy and these findings were validated in an independent cohort of secondary progressive (SPMS) patients, but not in a third cohorts of relapsing–remitting (RRMS) patients. Collectively, our analysis suggests that sphingomyelin-d18:1/14:0, mono-hexosylceramide-d18:1/20:0, and LPA-18:2 may represent important targets for future studies aimed at understanding disease progression in MS.

## Introduction

Multiple sclerosis (MS) is an inflammatory demyelinating disease of the central nervous system (CNS)^1^. Approximately 85% of patients with MS are characterized by a relapsing–remitting disease course (RRMS) in which acute neurological deficits are followed by spontaneous recovery^1^. In 65% of cases, RRMS patients develop secondary progressive MS (SPMS) in which disability accumulates over time without remission^1^. The remaining 15% have a progressive course of the disease from onset (primary progressive MS, PPMS)^1^. Although several anti-inflammatory and immunomodulatory drugs have been developed for the treatment of patients with RRMS, such treatments only show limited efficacy in progressive MS patients. Lipidomic and metabolomic approaches provide a powerful tool to identify molecules associated with pathological conditions, which may lead to the discovery of new therapeutic targets. Previous studies, mostly focused on RRMS, have reported differential lipids in the CSF and serum of MS patients compared with healthy individuals^2–6^. However, still very little is known about the lipid signals affecting the temporal progression of the disease course and this was the aim of our exploratory study. We analyzed plasma samples from healthy controls and from two groups of PPMS patients, defined by clinical and imaging parameters, as rapidly (PPMS-progressive, PPMS-P) or less rapidly (PPMS-non-progressive, PPMS-NP) progressing over time.

The detection of lipid species with differential abundance among the three groups were correlated with clinical and MRI signs of disease activity. Based on the small group size of our study in PPMS patients, we interrogated additional plasma lipidomic datasets, from two independently recruited cohorts of RRMS and SPMS patients with rapid or stable disease course, for validation of the main findings.

Overall, we identify decreased levels of specific membrane lipids with signaling capabilities in patients with a rapid disease course and suggest that further investigations on their signaling pathways may contribute to elucidating potential mechanisms underlying disease progression in MS.

## Results

**Figure 1:**
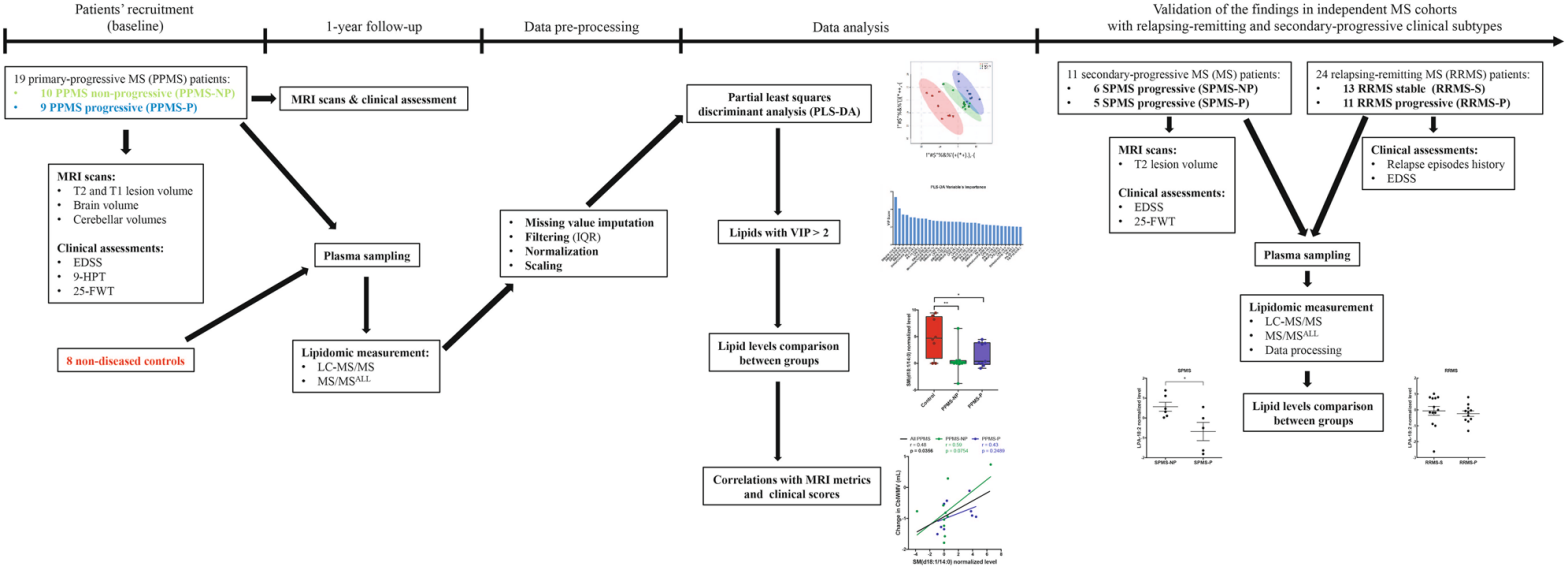
Flow chart of study procedures. The figure shows the overall study design. From left to right: recruitment of PPMS patients in the discovery cohort, MRI and clinical assessment at baseline, and MRI evaluation, clinical assessment and plasma collection at the one year follow up. Plasma samples were then subjected to LC–MS/MS and MS/MS^ALL^, followed by lipidomic data pre-processing. The PLS-DA was used to identify lipids driving separations between groups and those with a high VIP score (> 2.0) were evaluated further with statistical tests to identify differentially abundant lipids correlating with rapid disease progression. Two independently collected plasma lipidomic datasets from two distinct cohorts of SPMS and RRMS patients were similarly analyzed to validate the findings in PPMS patients also in the relapsing–remitting and secondary-progressive MS clinical subtypes.

**Figure 2:**
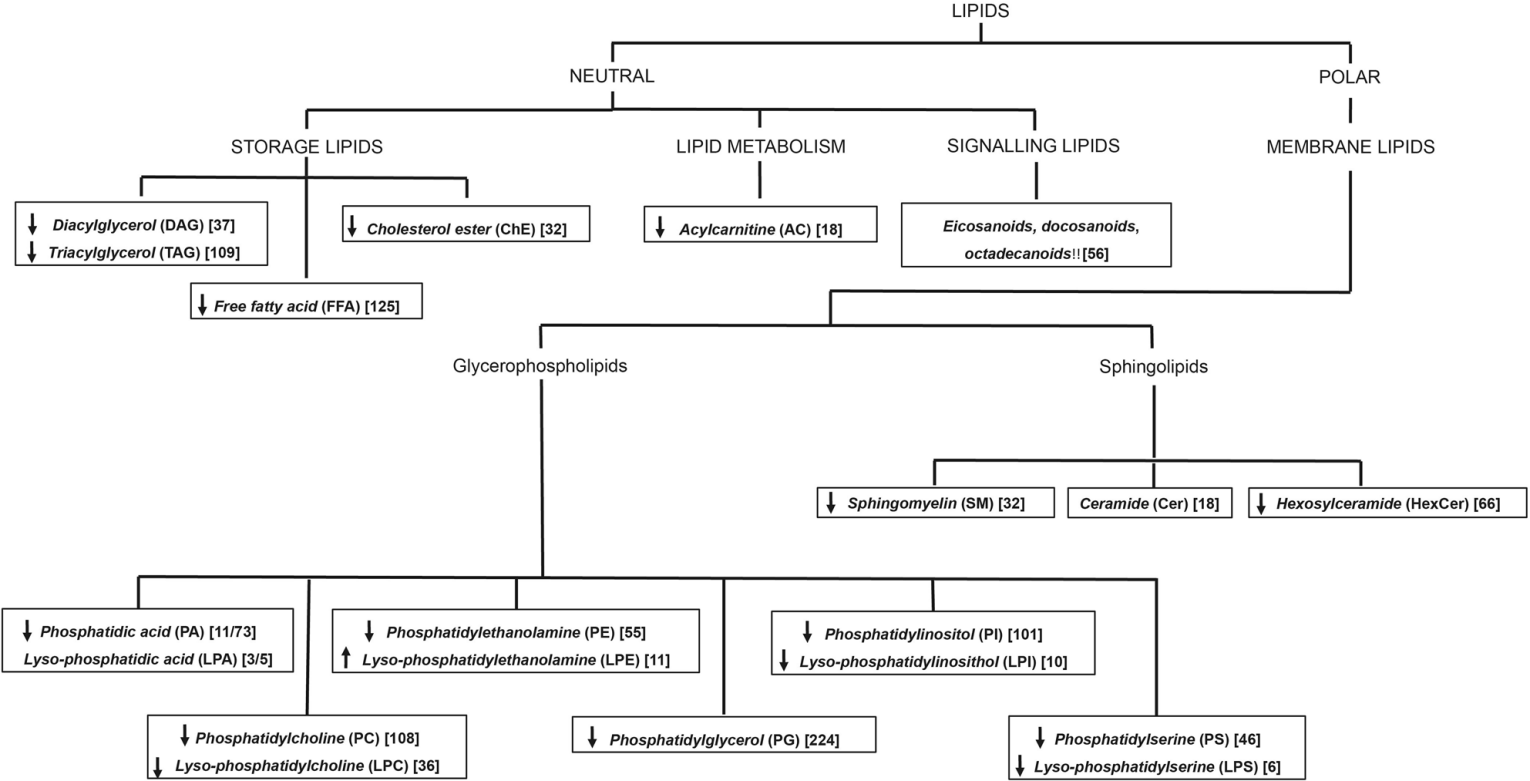
Graph shows the lipid classes detected in the plasma of patients with PPMS and healthy controls. Neutral and polar lipids were measured, and then classified based on their main biological function: storage, signalling, metabolism product, and membrane lipids. 1,068 lipid species, belonging to 15 different lipid classes, were detected in the plasma of PPMS patients. In bold-italic is indicated the name of the lipid class and in round brackets its abbreviation. In square brackets is indicated the total number of lipids detected per each class. Up-facing and down-facing arrows indicates fold-change (PPMS/controls) trends of increased or decreased lipid levels respectively.

Nineteen patients with a diagnosis of PPMS were sorted into distinct groups based on whether they experienced a decline of the neurological function and/or had the appearance of new MRI-visible T2 lesion(s) within one-year from enrolment (respectively PPMS-NP, n = 10; and PPMS-P, n = 9). Eight non-diseased controls were included for comparison. The demographic characteristics of the participants in the study are provided in Table 1, while the overall study design is illustrated in Fig. 1. PPMS-P patients were characterized by a significant exacerbation of neurological deficits as for the Expanded Disability Status Scale (EDSS) at the one-year follow-up (Table 2). PPMS patients were characterized by reduced cerebellar volume (CblV), cerebellar grey matter volume (CblGMV), and cerebellar white matter volume (CblWMV) at the one-year follow-up, no changes were observed in T2 lesion volume and brain volume (computed as percentage brain volume change, PBVC) (Tables 3 and 4). Consistent with our previous findings, suggesting a role for cerebellar volumes as short-term imaging metrics to monitor the disease progression in PPMS patients^7^, PPMS-P patients experienced a greater, significant reduction of CblV and CblGMV compared to PPMS-NP patients (Table 4). Blood was collected from fasting non-diseased controls and PPMS patients at the time of the one-year follow-up and processed for lipidomic analysis using a combination of liquid chromatography tandem mass spectrometry (LC–MS/MS, for signaling lipids) and direct infusion MS/MS^ALL^ analysis. In total, 1889 lipid species, belonging to 15 different lipid classes, were detected, of which 1,068 (57%) passed the quality control for above limit of quantitation. Lipids were divided into neutral and polar classes and further sub-classified based on their main biological function as: storage, structural, signaling or metabolic by-products (Fig. 2). The overall abundance of each lipid class in PPMS patients compared to controls is summarized in Fig. 2.

**Table 1:** Demographic features of patients and controls.

			PPMS				
	Controls	PPMS	PPMS-NP	PPMS-P	*p*-value^a^	*p*-value^b^	*p*-value^c^
Sample size	8	19	10	9	N/A	N/A	N/A
Age (range)	42.1 ± 8.4 (34–53)	49.8 ± 11.1 (32–65)	48.2 ± 10.4 (32–61)	51.7 ± 12.2 (33–65)	0.163	0.408	0.114
Sex	4 M/4F	8 M/11F	2 M/8F	6 M/3F	1.0	0.321	0.637
Disease duration, years	N/A	8.8 ± 5.1	9.3 ± 6.5	8.2 ± 3.1	N/A	N/A	N/A

PPMS, primary progressive MS; PPMS-NP, primary progressive MS with non-progressed disability; PPMS-P, primary progressive MS with progressed disability; M, male; F, female.

^a^PPMS versus controls.

^b^PPMS-NP versus controls.

^c^PPMS-P versus controls.

Mann–Whitney test was applied to assess differences in terms of age and disease duration.

Fisher exact test was applied to assess differences in terms of gender.

**Table 2:** Clinical characteristics of patients.

			PPMS			
	PPMS		PPMS-NP		PPMS-P	
	Baseline	1-Y FU	Baseline	1-Y FU	Baseline	1-Y FU
EDSS median	4.0 (1.5–6)	4.0 (2–6)	4.0 (1.5–6)	4.0 (2–6)	4.0 (2–6)	6.0* **(2.5–6)**
9-HPT dominant hand, seconds	31.5 (19.1–62.0)	34.6 (17.6–86.8)	31.7 (20.7–62.0)	37.1 (17.6–86.8)	31.3 (19.1–62.0)	31.0 (18.2–43.4)
9-HPT non-dominant hand, seconds	35.9 (21.0–82.8)	37.3 (21.4–143.1)	30.2 (21.0–40.3)	31.8 (21.4–43.5)	41.5 (24.9–82.8)	43.35 (25.0–143.1)
25-FWT, seconds	7.0 (3.9–12.4)	7.2 (4.2–12.2)	6.8 (4.3–10.7)	6.7 (4.2–10.1)	7.3 (3.9–12.4)	7.7 (4.5–12.2)

Statistically significant difference between groups (*p* < 0.05) is highlighted in bold.

1-Y FU = 1-year follow-up; EDSS = Expanded Disability Status Scale; 9-HPT = 9-Hole Peg Test; 25-FWT = 25-Foot Walking Test. Unless specified all values are express as mean (range).

*p < 0.05, Wilcoxon signed rank test for paired samples.

**Table 3:** MRI metrics at baseline and follow-up.

				PPMS					
	PPMS			PPMS-NP			PPMS-P		
	Baseline	1-Y FU	*p*-value^a^	Baseline	1-Y FU	*p*-value^a^	Baseline	1-Y FU	*p*-value^a^
T1 lesion	3.31 (± 5.41)	3.92 (± 6.61)	**0.005**	3.51 (± 6.68	4.49 (± 8.55)	**0.015**	3.08 (± 3.93)	3.27 (± 3.91)	0.139
T2 lesion	5.71 (± 7.79)	6.43 (± 8.55)	0.087	5.89 (± 9.62)	6.75 (± 10.15)	0.139	5.50 (± 5.68)	6.06 (± 6.94)	0.314
CblV	114.63 (± 13.16)	112.02 (± 12.62)	** < 0.001**	110.33 (± 14.57)	108.67 (± 14.77)	**0.005**	119.40 (± 10.12)	115.74 (± 9.15)	**0.008**
CblGMV	86.85 (± 9.33)	85.07 (± 8.62)	** < 0.001**	83.42 (± 9.35)	82.54 (± 9.29)	**0.013**	90.67 (± 8.13)	87.88 (± 7.28)	**0.008**
CblWMV	27.77 (± 5.48)	26.95 (± 5.36)	** < 0.001**	26.91 (± 6.66)	26.14 (± 6.56)	**0.017**	28.72 (± 3.98)	27.85 (± 3.80)	**0.008**

Statistically significant differences between groups (*p* < 0.05) are highlighted in bold.

^a^Wilcoxon signed rank test.

1-Y FU = 1-year follow-up; CblV, cerebellar volume; CblGMV, cerebellar grey matter volume; CblWMV, cerebellar white matter volume. All values are expressed in mL. Values represent mean (± SD).

**Table 4:** Delta comparison PPMS-NP versus PPMS-P.

	Delta PPMS-NP	Delta PPMS-P	*p*-value^a^
T1 lesion (mL)	0.98	0.19	0.356
T2 lesion (mL)	0.86	0.57	0.842
PBVC (mL)	− 0.40	− 0.89	0.780
CblV (mL)	− 1.66	− 3.66	**0.008**
CblGMV (mL)	− 0.89	− 2.78	**0.008**
CblWMV (mL)	− 0.77	− 0.87	1

Statistically significant differences between groups (*p* < 0.05) are highlighted in bold.

^a^Mann–Whitney U test for delta PPMS-NP versus PPMS-P.

PBVC, percentage brain volume change; CblV, cerebellar volume; CblGMV, cerebellar grey matter volume; CblWMV, cerebellar white matter volume. Delta are computed as follow-up values minus baseline values.

### Analysis of plasma lipidomic identifies lipids with differential abundance in the plasma of progressive patients

**Figure 3:**
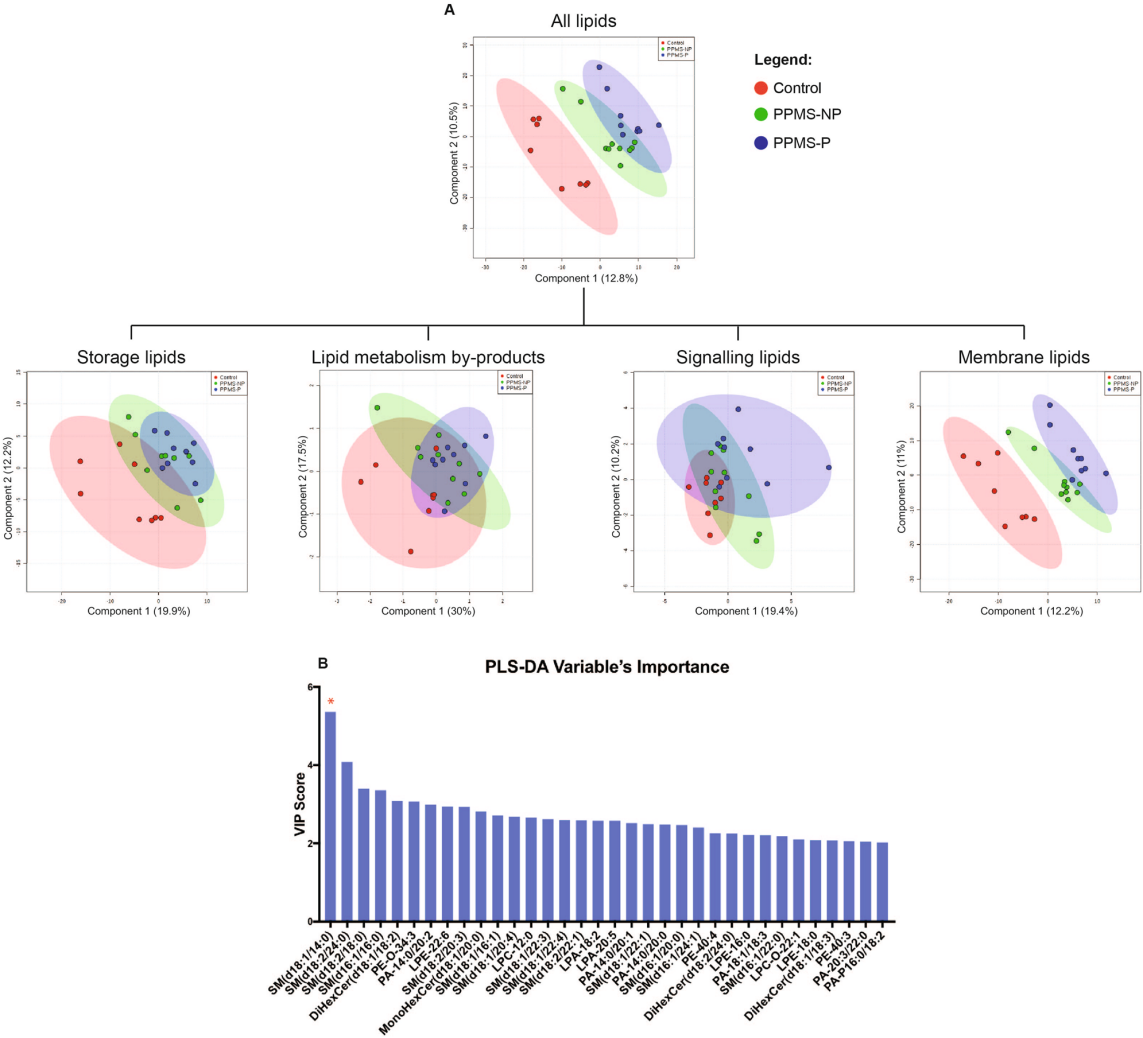
PLS-DA discriminates PPMS patients from non-diseased controls based on membrane lipid levels in the plasma. (**A**) The plots show separation of controls, PPMS-P, and PPMS-NP patients based on the first two components of the partial least square discriminant analysis (PLS-DA). PLS-DA was performed using normalized lipid levels measured in the plasma. Storage, signaling lipids, and lipid metabolism by-products (acyl-carnitines) alone were not able to separate the three groups at the PLS-DA. By contrast, membrane lipids were sufficient to efficiently discriminate patients from controls and PPMS-P from PPMS-NP patients. Control, n = 8; PPMS-NP, n = 10; PPMS-P, n = 9. (**B**) Graph shows the Variable’s Importance PLS-DA (VIP) scores of lipids that more prominently contributed to the PLS-DA model (VIP > 2). Red asterisk indicates SM(d18:1/14:0), the lipid with the highest VIP score and thus one of the lipids that most contributed to the PLS-DA model.

We performed a partial least squares discriminant analysis (PLS-DA) to evaluate lipids with differential abundance in the plasma of PPMS patients and controls (Fig. 2 and Supplementary Fig. S1). First, we included the entire lipid dataset in the PLS-DA model, which efficiently differentiated patients from controls, and progressive patients with rapid (progressed disability) from slower (without progressed disability) disease course (Fig. 3A). A similar analysis performed using only values from the membrane lipids—but not using lipid metabolism by-products (acyl-carnitines), storage, or signaling lipids alone—led to a well-defined separation among the three groups (Fig. 3A). These findings suggested that in PPMS patients the differential abundance of membrane lipids is more prominent than other lipid categories and may contribute in identifying patients from healthy individuals, while providing interesting lipid targets to be further explored in the study of disease progression. To assess the reliability of the model in separating the groups using membrane lipids, we calculated the model-fit metrics for the first five components of the PLS-DA (Supplementary Figure S1). The membrane lipids driving the separation between groups were identified using the variable’s importance PLS-DA (VIP) score (VIP > 2). Sphingomyelin SM(d18:1/14:0) was the lipid with the highest VIP score (Fig. 3B), thus contributing the most to the PLS-DA model. Consistent with previously published lipidomic studies in MS^3,8^, we further evaluated the lipids with high VIP with more robust statistical analyses, to define the ones with differential abundance and then asked whether they correlated with MRI and/or clinical signs of disease progression. Thus, from hereafter we will focus only on the differential membrane lipids identified by our analysis.

### Lipidomic changes in the plasma of patients with PPMS

**Figure 4:**
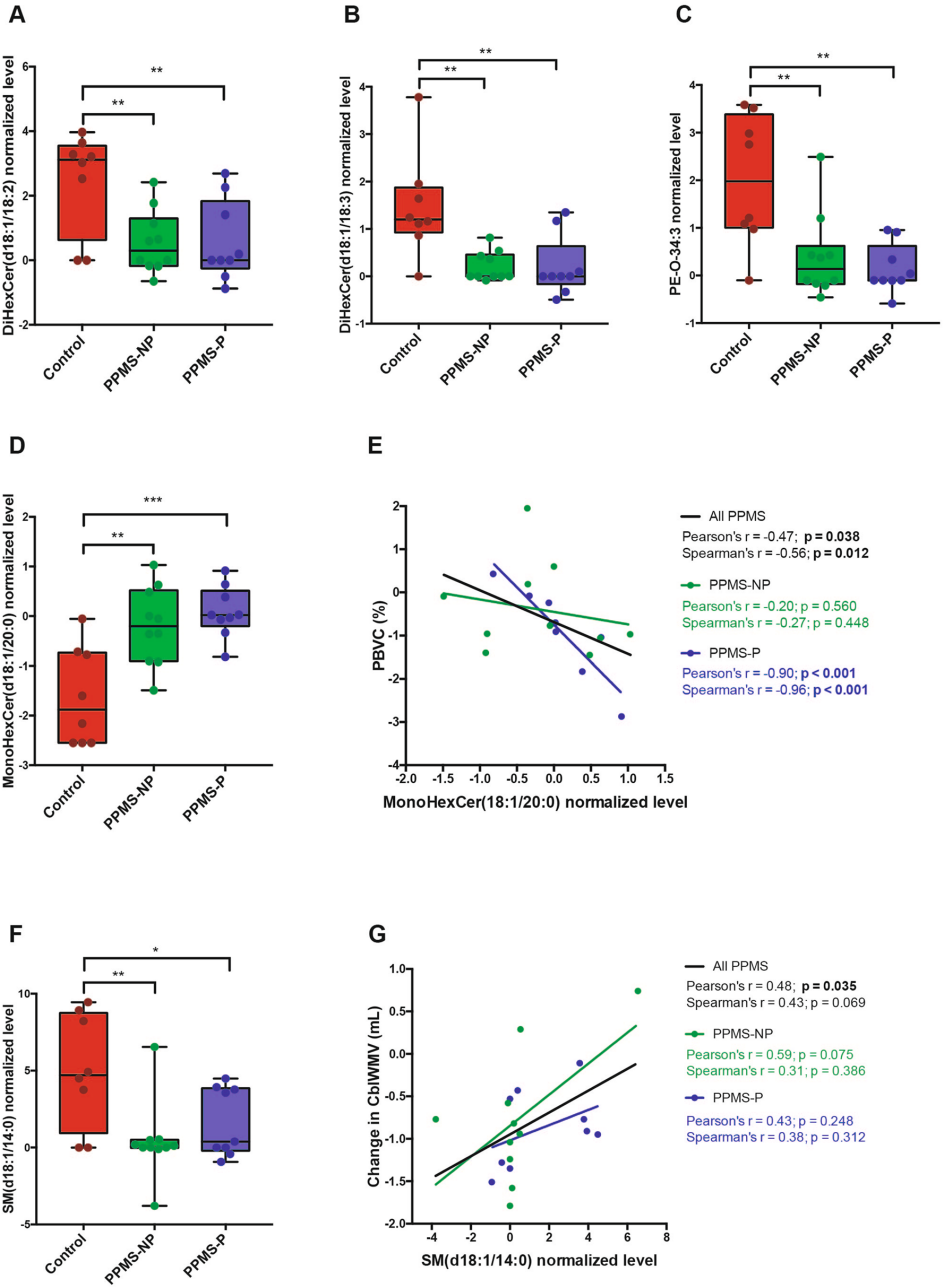
Lipids differentially abundant in the plasma of PPMS patients compared with controls. (**A-D,F**) Graphs show normalized levels of differentially abundant lipids in the plasma of healthy controls, PPMS-NP and PPMS-P patients. The levels of (**A**) Di-hexosylceramide(d18:1/18:2) [DiHexCer(d18:1/18:2)], (**B**) DiHexCer(d18:1/18:3), (**C**) phosphatidylethanolamine-O-34:3 (PE-O-34:3), and (**F**) sphingomyelin SM(d18:1/14:0) were significantly lower in the plasma of PPMS patients compared with controls, while the levels of (**C**) mono-hexosylceramide(d18:1/20:0) [MonoHexCer(d18:1/20:0)] were significantly higher. Boxes indicate the interquartile range, horizontal lines indicate group medians, whiskers connect the lowest and the highest observations. The statistical analysis was performed using the One-way ANOVA with Dunnet’s multiple comparison test (*p < 0.05, **p < 0.01, ***p < 0.001). Control, n = 8; PPMS-NP, n = 10; PPMS-P, n = 9. (**G** and** F**) Graphs show correlations between normalized plasma lipid levels of (**E**) MonoHexCer(d18:1/20:0) and (**G**) SM(d18:1/14:0) with changes in MRI metrics at the 1-year follow-up. Changes in MRI volumes were computed as follow-up values minus baseline values. (**E**) Higher abundance of MonoHexCer(d18:1/20:0) in the plasma of PPMS patients correlated with more severe brain volume loss (calculated as percentage brain volume change, PBVC). (**G**) Higher levels of SM(d18:1/14:0) correlated with less severe loss of CblWMV. Lines represent the linear regressions of all patients (black), PPMS-NP (green), and PPMS-P (blue) subgroups. Correlations were assessed using both the Pearson and Spearman methods. R-coefficient (r) and p*-*values (p) are indicated in black for the entire PPMS population, in green and blue for the PPMS-NP and PPMS-P group respectively. *P* < 0.05 (in bold) was considered significant. PPMS-NP, n = 10; PPMS-P, n = 9.

In order to determine whether plasma lipids of PPMS patients differed from controls, we focused on lipids with differential abundance in the plasma of non-diseased controls, compared to PPMS patients regardless of the progressed disability status. Five lipid species, belonging to 3 different classes, were identified as differentially abundant. Among these lipids 3 were hexosylceramides (HexCer) (Fig. 4A,B,D), a phosphatidylethanolamine (PE) (Fig. 4C), and a sphingomyelin (SM) (Fig. 4F). The long-chain mono-hexosylceramide, MonoHexCer(d18:1/20:0) was more abundant in the plasma of PPMS patients compared with controls (Fig. 4D), its levels were higher in the plasma of those patients with increased brain atrophy at the 1-year follow-up (computed as percentage brain volume change, PBVC), and they correlated more strongly with PBVC values in PPMS-P patients (Fig. 4E). This finding supports previous studies on the importance of this lipid in neurodegeneration^9^. The remaining two hexosyl-ceramides [DiHexCer(d18:1/18:2), and DiHexCer(d18:1/18:3)] were less abundant in PPMS patients compared to controls (Fig. 4A,B) and their levels did not correlate with clinical and MRI signs of disease activity. SM(d18:1/14:0) was significantly decreased in the plasma of PPMS patients compared with controls (Fig. 4F) and a positive correlation was found between its levels and MRI metrics of decreased CblWMV at the one-year follow-up. Notably, high levels of SM(d18:1/14:0) in the plasma, corresponded to a smaller reduction of the CblWMV after one year (Fig. 4G). These findings suggest that SM(d18:1/14:0) levels may reflect an overall preservation of neuro-glial function in PPMS patients.

### Lipid species related to progressed disability

**Figure 5:**
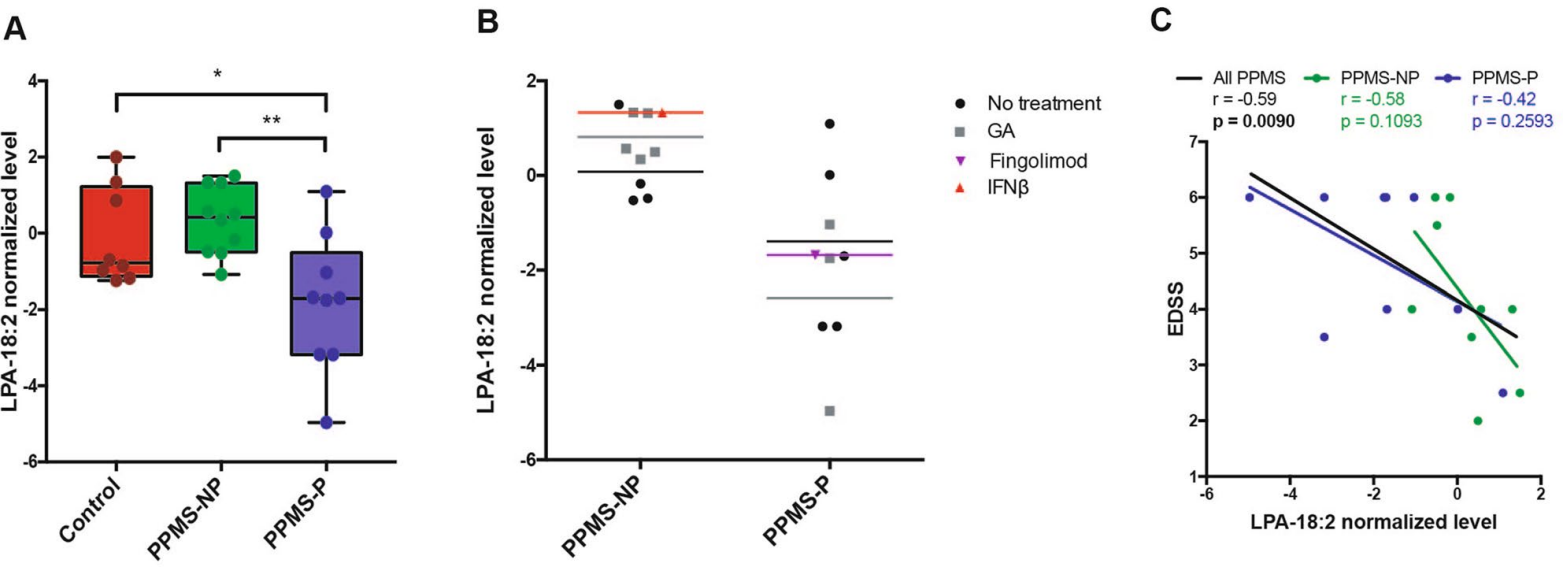
The lower plasma levels of LPA 18:2 in PPMS-P patients compared to PPMS-NP, are detected independently of the use of immunomodulatory drugs and correlate with severe neurological deficits. (**A**) Graph shows normalized levels of lysophosphatidic acid 18:2 (LPA-18:2) in the plasma of PPMS-NP and PPMS-P patients and non-diseased controls. LPA-18:2 levels were significantly lower in samples from PPMS-P patients than PPMS-NP and non-diseased controls. No significant differences were detected between PPMS-NP and controls. Boxes indicate the interquartile range, horizontal lines indicate group medians, whiskers connect the lowest and the highest observations. The statistical analysis was performed using the One-way ANOVA with Tukey’s multiple comparison test (*p < 0.05, **p < 0.01). Control, n = 8; PPMS-NP, n = 10; PPMS-P, n = 9. (**B**) Graph shows LPA-18:2 levels in the plasma of untreated and treated PPMS-NP and PPMS-P patients. Untreated: PPMS-NP, n = 4; PPMS-P, n = 5; treated with glatiramer acetate (GA): PPMS-NP, n = 5; PPMS-P, n = 3; treated with Interferon-β (IFNβ): PPMS-NP, n = 1; treated with Fingolimod: PPMS-P, n = 1. Color coded horizontal lines depict group means. (**C**) LPA-18:2 levels in the plasma inversely correlated with the severity of the neurological deficit assessed using the EDSS method. Green and blue dots show the values of PPMS-NP and PPMS-P patients respectively. Lines represent the linear regressions of all patients together (black), PPMS-NP (green), and PPMS-P (blue) subgroups. Correlations were computed using the Spearman’s r coefficient. *p* < 0.05 (in bold) was considered significant. PPMS-NP, n = 10; PPMS-P, n = 9.

**Figure 6:**
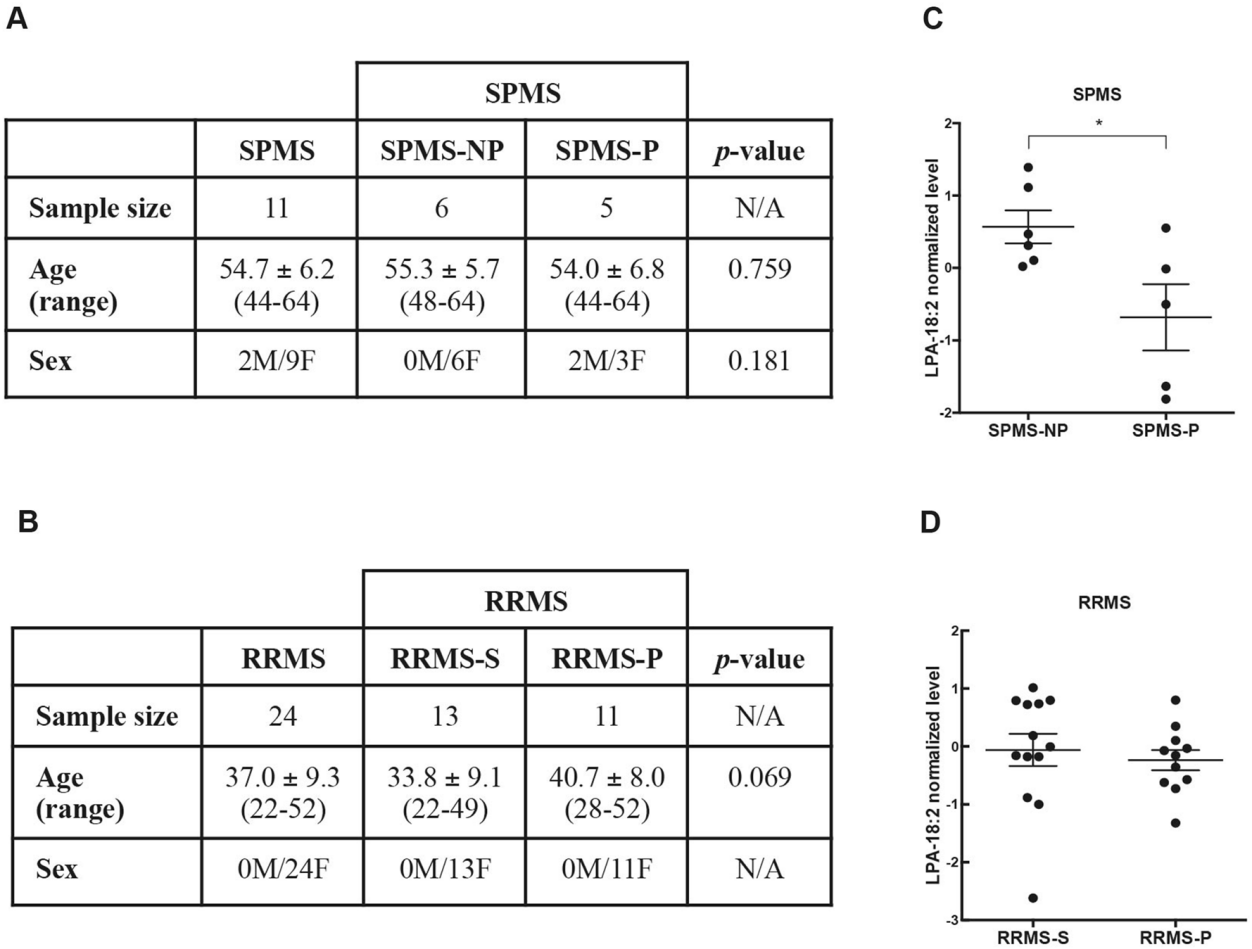
LPA-18:2 levels in RRMS and SPMS patients with stable or progressed disability. (**A**,**B**) Tables show the demographic features of the cohorts of SPMS (**A**) and RRMS (**B**) patients from which additional plasma lipidomic datasets were obtained. SPMS, secondary progressive MS; SPMS-NP, secondary progressive MS with non-progressed disability; SPMS-P, secondary progressive MS with progressed disability; RRMS, relapsing–remitting MS; RRMS-S, relapsing–remitting MS with stable disability; RRMS-P, relapsing–remitting MS with progressed disability; M, male; F, female. Age is expressed as mean ± standard deviation. Mann–Whitney test was applied to assess differences in terms of age. Fisher exact test was applied to assess differences in terms of gender. (**C,D**) Graphs show LPA-18:2 levels in the plasma of SPMS and RRMS patients with either a stable or more rapid disease course. Scatter plot graphs are presented with the mean ± SEM. (**C**) LPA-18:2 levels were lower in the plasma of SPMS-P compared with SPMS-NP patients. SPMS-NP, n = 6; SPMS-P, n = 5. (**D**) No differences in LPA-18:2 plasma levels were observed between RRMS-S and RRMS-P patients. RRMS-S, n = 13; RRMS-P, n = 11. Differences in LPA-18:2 levels were assessed using the independent t-test (*p < 0.05).

Further interrogation of the lipidomic datasets to define lipids with differential abundance between PPMS-P and PPMS-NP patients, identified lysophosphatidic acid 18:2 (LPA-18:2). This lipid was less abundant in the plasma of PPMS-P patients compared to PPMS-NP patients and controls and therefore was considered an interesting candidate for further analysis (Fig. 5A). Since patients in the discovery cohort were not stratified by therapy, the question remained on the putative effect of disease modifying drugs on the lipid levels. For this reason, we plotted the LPA-18:2 levels in all the patients including their current treatment at time of analysis and asked whether differences could be detected between untreated patients versus those on specific immunomodulators (Fig. 5B). Interestingly, the average levels of LPA-18:2 in the plasma were consistently lower in patients with a rapid disease course (PPMS-P) compared with those with a slower course (PPMS-NP) and this effect was detected regardless of therapy (Fig. 5B). Notably, LPA-18:2 levels inversely correlated (r = − 0.59; *p* = 0.009) with the severity of the neurological deficits (Fig. 5C) with higher LPA-18:2 levels detected in PPMS patients with milder neurological dysfunction. To provide an independent validation for the levels of LPA-18:2, we interrogated two distinct plasma lipidomic datasets from two independent validation cohorts of SPMS patients (unpublished) and RRMS^10^. The first cohort included patients with progressive (SPMS-P, n = 5) or non-progressive (SPMS-NP, n = 6) disease course (Fig. 6A; Supplementary Table S1). All SPMS patients were treated with dimethyl fumarate (Tecfidera, Biogen) for one-year prior the lipidomic analysis. Consistent with our finding in the PPMS cohort, LPA-18:2 levels were significantly lower in the plasma of SPMS-P compared with SPMS-NP patients, regardless of therapy (Fig. 6C). The second cohort included untreated recently diagnosed RRMS patients^10^ (Fig. 6B; Supplementary Table S2) with a stable (RRMS-S, n = 13) or a more severe (RRMS-P, n = 11) disease course at the 1 year follow up. No differences in LPA-18:2 plasma levels were detected between RRMS-S and RRMS-P groups (Fig. 6D) and no correlations were found between LPA-18:2 levels and the EDSS score in both the SPMS and RRMS cohorts (data not shown). Although we recognize the small sample size of this study, the detection of lower LPA18:2 levels in two independent cohorts of patients with progressed disability, compared with patients with non-progressed disability, raises the possibility that LPA-18:2 may be a lipid mediator whose signaling pathway may be worth studying to elucidate mechanisms of disease exacerbation in the progressive forms of MS.

## Discussion

Lipidomic approaches have been employed to identify lipid signatures that can potentially lead to the discovery of biomarkers, as well as provide novel information about pathological mechanisms involved in MS^11^. Recent studies have investigated whether changes in lipid levels occur in the serum of patients with MS^3–5^. Teunissen (2003), and Del Boccio (2011) performed targeted lipidomic analyses to examine the relative abundance of specific lipid classes (cholesterols, and phosphatidylcholines respectively). Similar to our study, Villoslada (2017) performed a global lipidomic analysis. Those studies focused on determining the overall imbalance of lipid classes between patients and controls, and primarily included patients with RRMS, with the inclusion of few PPMS patients. Our study on a small cohort of PPMS patients with distinct disease course of progression, in contrast, was intended as exploratory, to identify potential lipid molecular species that would differ between PPMS patients with progressed and non-progressed disability. A PLS-DA analysis allowed to identify the lipid classes whose overall levels could help in discriminating patients from healthy individuals and, within these lipid classes, the molecular species contributing to the clustering. We did not observe changes in the overall levels of plasma signaling lipids (eicosanoids, docosanoids, and octadecanoids), although those lipids play critical roles in the regulation of many inflammatory processes including MS^12,13^. In contrast, a trend towards reduced levels of storage lipids (fatty acids, cholesterol esters, triglycerides, and diglycerides) was observed in the plasma of patients with PPMS. However, storage lipid levels alone did not classify participants as healthy individuals or PPMS patients at the PLS-DA model. Nonetheless, we believe that storage and diet-derived lipids may inform on the pathology of the disease and therefore worth of further investigation.

Interestingly, among all lipids analyzed, the levels of membrane lipids (phospholipids and sphingolipids) were remarkably altered in PPMS patients compared with non-diseased individuals, and their levels were sufficient to discriminate patients from controls at the PLS-DA. Importantly, the computational model also separated PPMS-NP from PPMS-P patients, suggesting that membrane lipids may vary in relation to the disease activity and could represent lipid mediators affecting disease progression, although this concept deserves careful future investigation. Demyelination has been commonly thought to increase the level of membrane lipids in the peripheral circulation via the leakage of myelin-derived lipids through the damaged blood–brain barrier. However, this concept was not supported by experimental evidence, showing an overall reduction of many membrane lipids in the blood of patients with MS^3,4^. A potential explanation for this finding is the reduction of lipid synthesis in the CNS consequent to the slow demyelinating process and the progressive replacement of lipid-rich tissue (myelin) with glial scars enriched in astrocytes and connective tissue. Nonetheless, MonoHexCer(d18:1/20:0), a long-chain ceramide of cell membranes and myelin sheaths, was significantly increased in the plasma of PPMS patients compared with non-disease controls. Interestingly, its plasma levels correlated with increased brain atrophy at the one-year follow-up and more prominently in patients with progressed disability. Possibly, MonoHexCer(d18:1/20:0) levels in the plasma may depict more sensitively the ongoing myelin and cellular damage affecting the CNS of patients with MS.

SM(d18:1/14:0) was the lipid that most strongly contributed to the separation of the three groups at the PLS-DA model and was significantly decreased in PPMS patients compared with controls. SM(d18:1/14:0) levels in the plasma correlated with greater changes in cerebellar white matter volume at the one-year follow-up. Sphingomyelin is an important components of plasma membranes, of which the brain is particularly enriched. Furthermore, increasing evidence suggests signaling functions of short-chain sphingomyelins in the regulation of many intracellular processes, raising the possibility that SM(d18:1/14:0) may contribute to signaling pathways exerting a neuroprotective effect.

We also aimed to identify those lipids that were differentially abundant in the plasma of PPMS with progressed disability compared with those with non-progressed disability. In our cohort, only LPA-18:2 was differentially abundant and significantly decreased in the plasma of PPMS-P patients. A similar finding was detected in an independent cohort of SPMS patients, with lower LPA-18:2 levels in the plasma of patients with rapidly deteriorating disease course compared to those with a slower progression.

LPAs were initially considered inert intermediates in the biosynthesis of membrane lipids, and hydrolysis products of phosphatidic acid. However, recent evidence has identified LPAs as multifunctional bioactive lipids with signaling function^14^. In the CNS, LPA-mediated signaling can influence a variety of neural processes including but not limited to oligodendrocyte maturation, neuronal plasticity and synaptic connections^15^. Thus, decreased levels of LPA-18:2 in the plasma of PPMS-P patients may be related to neurodegeneration and decline in oligodendrocyte homeostasis, while the high levels of LPA-18:2 in patients with a milder disease course may be attributed to a compensatory mechanism activated by this lipid and intended to preserve, at least in part, neuronal health.

Alternatively, a recent study^16^ suggested a role for serum LPAs in the resolution of the inflammatory response in RRMS patients and mice with experimental autoimmune encephalomyelitis (EAE, a model of MS). Interestingly, in their study, Schmitz and colleagues (2017) found LPA levels to inversely correlate with disease activity both in EAE models and in RRMS patients^16^. Our data in recently diagnosed RRMS patients did not support this interpretation, however, we cannot exclude the possibility that differences in LPA-18:2 levels may be identified in RRMS patients with longer disease duration.

Overall, this study provides useful insights on lipid dysregulation in patients with a rapidly progressing disease course and identifies molecular targets which may be considered for future investigations on mechanisms underlying the temporal progression of the disease course in MS.

## Materials and methods

### Patients

The institutional review board of Icahn School of Medicine at Mount Sinai (New York, NY) approved the study and all participants gave written informed consent before investigation according to the Declaration of Helsinki. All subjects underwent blood draw in fasting conditions, and clinical and MRI assessment on the same day. The BioMed IRB approved the study at the MS Center of Northeastern New York from where the data for the SPMS cohort were collected. Procedures were the same as at the Icahn School of Medicine at Mount Sinai other than there was a 7 days window for MRI assessment.

#### PPMS discovery cohort

Twenty-six patients who met the modified McDonald diagnostic criteria and presented a primary progressive course of MS (PPMS) together with twenty age and gender matched non-diseased controls were enrolled in the study. Of these participants, nineteen patients and eight non-diseased controls for which blood sample was available entered the final analysis (Table 1). Inclusion criteria for PPMS patients were: (a) age between 25 and 65 years old, (b) expanded disability status (EDSS) lower than 6.5 at the time of recruitment, (c) disease duration up to 15 years. In the absence of exceptional events, patients were visited every 6 months from the first screening (baseline). The use of immunomodulatory drugs was allowed, but treated patients had to be on current treatment for at least 1-year. Of the recruited patients, nine were not on any treatment, while ten were under immunomodulatory therapy (eight patients were using glatiramer acetate, one patient interferon beta-1a, and one fingolimod).

Clinical disability and disease progression over time was assessed as previously described^7^. Briefly, clinical disability was evaluated with the EDSS, 25-foot walk and 9-hole-peg tests (25-FWT and 9-HPT) at baseline, month six and month twelve. To confirm sustained progression, patients were further assessed during a clinical follow-up visit 12 months after study termination. Clinical worsening was defined as EDSS score increase of one point if the baseline EDSS score was less than or equal to five, or an increase of 0.5 if it was greater than five or change of > 20% for 25-FWT or change of > 20% for 9-HPT scores. MRI worsening was defined by the presence of disease activity at month 6 or 12 compared with baseline (i.e. presence of new brain or spine lesions on T2-weighted images). Sustained progression was defined as (A) clinical worsening (i) at month 6 compared with baseline, confirmed at month 12 or (ii) at clinical follow-up visit 12 months after study termination compared with month 12; (B) MRI occurrence of a new T2 visible lesion.

#### RRMS validation cohort

Twenty-four patients who presented a relapsing–remitting disease course of MS (RRMS) were classified to have a progressive course of the disease (RRMS-P, n = 11) if they had 1) at least one relapse episode and/or 2) an increase ≥ 0.5 of the EDSS score over a one-year follow-up period, confirmed at the two-year follow-up visit. Otherwise, patients were considered “stable” (RRMS-S, n = 13).

#### SPMS validation cohort

Eleven patients with secondary progressive MS (SPMS) as per the modified McDonald diagnostic criteria were classified to have a more rapid (SPMS-P, n = 5) or slower (SPMS-NP, n = 6) disease course based on clinical and MRI signs of sustained disability. Sustained progression was defined, as described previously, as (A) clinical worsening (i) at month 6 compared with baseline, confirmed at month 12 or (ii) at clinical follow-up visit 12 months after study termination compared with month 12^7^; (B) increase (≥ 5%) at the MRI T2 lesion volume. Clinical worsening was defined as EDSS score increase of one point if the baseline EDSS score was less than or equal to five, or an increase of 0.5 if it was greater than five or change of > 20% for 25-FWT^7^. All patients in this cohort were under treatment with dimethyl fumarate (Tecfidera, Biogen) for the one-year prior the lipidomic analysis.

### MRI acquisition and analysis

MRI was performed using a 3.0 T scanner (Philips Achieva, The Netherlands) with an 8-channel SENSE phased-array head coil and a 16-channel receive-only neuro-vascular spine coil. The MRI protocol included a brain axial dual echo TSE and a brain sagittal 3D T1-weighted turbo field echo sequences as previously described^7^. The spine sagittal T2- weighted TSE sequence was acquired as following: TR = 4,097 ms, TE = 120 ms, FOV = 250 × 250 mm, matrix size = 512 × 512, 13 3 mm-thick slices. Brain T2 and T1 lesion volumes (LV) were measured as described before^17^. Cerebellar lesion loads were measured on dual echo/3D T1 scans following the same steps applied for whole brain LV quantification. Two time-point percentage brain volume change (PBVC) was computed with SIENA^18^ on T1-weighted-lesion-filled and non-uniformity corrected images. Cerebellar cortical and WM volumes were measured using Freesurfer v5.3.0 longitudinal pipeline^19,20^.

### Lipidomic analysis

Plasma was purified from ~ 10 mL of blood collected from consented patients and non-diseased controls in fasting conditions. Blood was centrifuged at 400 rcf for 20 min, isolated, and further purified by centrifugation at 1,500 rcf for 15 min prior to being aliquoted and stored at − 80 °C. Lipidomic analyses were performed by BERG LCC (Framingham, MA) using liquid chromatography tandem mass spectrometry (LC–MS/MS) and direct infusion shotgun lipidomics (MS/MS^ALL^) as described in the supplementary methods. Briefly, a mixture of deuterium-labeled and odd chain phospholipid standards, chosen to represent each lipid class, was added to plasma. Following lipid separation, mass spectrometry measurement of signalling lipids was performed on a SCIEX TripleTOF 6600 system using the MRMHR strategy. Acylcarnitines, storage and membrane lipids were measured using a customized data-independent analysis strategy on the TripleTOF 5,600^+^ as previously described^21^. Lipid levels were determined measuring spectra peak areas relative to internal standards using an in-house library on MultiQuant software (SCIEX). After variance stabilization, each sample was median centered and log transformed (referred simply as “normalized lipid levels”), reflecting lipids relative abundances among samples and not actual concentrations. Lipids that were below the detection level in the majority of samples, as well as outliers, were filtered out using the interquartile range (IQR) method in Metaboanalyst v3.0 (https://www.metaboanalyst.ca).

### PLSD-DA analysis

The PLS-DA was performed in Metaboanalyst v3.0 (https://www.metaboanalyst.ca) to assess whether lipid levels can discriminate PPMS-P and PPMS-NP patients from non-diseased controls and to identify those lipids that drive the separation. We validated the PLS-DA using the tenfold cross validation method reporting the model performance overview for the first five components of the PLS-DA (Supplementary Figure S1). The PLS-DA showed satisfactory classification accuracy and high R2Y values (> 0.6) indicating the goodness of the model in assessing the degree to which the variables classify the different groups (Supplementary Fig. S1). The five components had low but positive Q2Y values (a quality assessment which estimates consistency between original data and predicted data estimated from cross-validation) indicating a moderate predictive ability of the model (Supplementary Fig. S1).

### Statistical analysis

All data were tested for Gaussian distribution using D’Agostino and Pearson omnibus normality test. Non-parametric or parametric statistical tests were used for comparison of not-normally or normally distributed datasets respectively. Mann–Whitney and Fisher exact tests in SPSS 22.0 (Chicago, IL) were applied to assess differences in terms of age and gender between patients and controls. Changes in clinical and MRI metrics over one-year follow-up were assessed with the Wilcoxon test using SPSS 22.0 (Chicago, IL). Partial least squares-discriminant analysis (PLS-DA) was performed using Metaboanalyst v3.0 (https://www.metaboanalyst.ca). Differentially abundant lipids between groups (control, PPMS-P and PPMS-NP) were detected using GraphPad Prism v7.0. The independent t-test was used when comparing only two groups. The One-way ANOVA was used to compare more than two groups as described in the figure legends. Briefly, the One-way ANOVA analysis with Dunnett’s multiple comparison post-test was applied to compare all groups to control, while the Tukey’s multiple comparison post-test was used to compare all groups to each other. The non-parametric Spearman’s method was used to detect correlations between lipid levels and non-linear variables (i.e. EDSS scores) and between normally distributed variables due to the small sample size of the groups. Pearson’s rank correlation was applied in addition to the Spearman’s method when data fulfilled the assumption of normality at the D’Agostino and Pearson omnibus normality test. All *p-*values are reported as two-sided significance levels and considered statistically significant when *p* < 0.05.

## Supplementary information

Supplementary information.

## Data Availability

The datasets generated and analyzed during the current study are available from the corresponding authors on reasonable request.
