# Supplementary material for: Retrospective unbiased plasma lipidomic of progressive multiple sclerosis patients-identifies lipids discriminating those with faster clinical deterioration
Source: Sci Rep. 2020 Sep 24;10:15644. doi: 10.1038/s41598-020-72654-8 (PMC7515876; doi:10.1038/s41598-020-72654-8)
Supplement: Supplementary file 1 — Supplementary information. [file 41598_2020_72654_MOESM1_ESM.docx]

**Retrospective unbiased plasma lipidomic of progressive multiple sclerosis patients**

**identifies lipids discriminating those with faster clinical deterioration**

Mario Amatruda^1,2*^, Maria Petracca^2,3^, Maureen Wentling^1^, Benjamin Inbar^1^, Kamilah Castro^1,4^, Emily Y Chen^5^, Michael A Kiebish^5^, Keith Edwards^6^, Matilde Inglese^2,7,8^, Patrizia Casaccia^1,4*^

^1^Advanced Science Research Center at the Graduate Center of the City University of New York, New York, NY, USA

^2^Department of Neurology, Icahn School of Medicine at Mount Sinai, New York, NY, USA

^3^Department of Neurosciences, Reproductive and Odontostomatological Sciences, Federico II University, Naples, Italy

^4^Department of Neuroscience, Graduate School of Biomedical Sciences, Icahn School of Medicine at Mount Sinai, New York, NY, USA

^5^BERG LLC, Framingham, MA, USA

^6^MS Center of Northeastern New York, Latham, NY USA

^7^Department of Radiology, Icahn School of Medicine at Mount Sinai, New York, NY, USA

^8^Department of Neurosciences, Rehabilitation, Ophthalmology, Genetics, Maternal and Child Health (DiNOGMI) and Center of Excellence for Biomedical Research (CEBR), Neurologic Clinic, University of Genoa, Genoa, Italy

**SUPPLEMENTARY METHODS**

**Lipidomic analysis**

Plasma was isolated and purified from approximately 10 mL of blood collected from consented patients and non-diseased controls in fasting conditions, at the Corinne Goldsmith Dickinson Center for Multiple Sclerosis at Mount Sinai (New York, NY). Blood was transferred in vials containing 5 mL histopaque-1077 (Sigma 10771) such that the blood-to-histopaque ratio was 1:1. Vials were then centrifuged at 400 rcf for 20 minutes producing a 4-way phase separation into plasma, buffy coat, histopaque, and erythrocytes. Plasma was collected and further purified by centrifugation at 1500 rcf for 15 minutes. The purified plasma supernatant was placed into final aliquots of 500 µL and stored at -80C. Lipidomic analyses were performed by BERG LCC (Framingham, MA). Lipid standards were purchased from Avanti Polar Lipids (Alabaster, AL), Nu-Chek Prep Inc. (Waterville, MN), Matreya (State College, PA), Cayman Chemical Company (Ann Arbor, MI), Sigma-Aldrich (St. Louis, MO), Santa Cruz Biotechnology (Dallas, TX), or Cambridge Isotope Laboratories (Tewksbury, MA). All solvents were of HPLC or LC/MS grade and were acquired from Fisher Scientific (Waltham, MA) or VWR International (Radnor, PA). The measurement of signaling lipids (eicosanoids, docosanoids, and octadecanoids) was performed using an optimized protocol of liquid chromatography tandem mass spectrometry (LC-MS/MS) adapted from Powell *et al.* (1999)^1^. Briefly, a mixture of deuterium-labeled internal standards was added to aliquots of 100 µL of plasma, followed by 3x volume of cold methanol (Met-OH). Samples were vortexed for 5 min and stored at −20 °C overnight.  Cold samples were centrifuged at 14,000g at 4°C for 10 minutes, and the supernatant was then transferred to a new tube. Then, 3 mL of acidified H2O (pH 3.5) was added to each sample prior to C18 solid-phase extraction performed as previously described^1^. The methyl-formate fractions were collected, dried under nitrogen, and reconstituted in 50 µL Met-OH:H2O (1:1, v/v). Samples were transferred to 0.5 mL tubes and centrifuged at 20,000g at 4°C for 10 min. Thirty-five microliters of supernatant was transferred to LC–MS vials for analysis using the BERG LC–MS/MS mediator lipidomics platform.  Separation of eicosanoids was performed on an Ekspert MicroLC 200 system (Eksigent Technologies) with a Synergi Fusion-RP capillary C18 column (150 × 0.5 mm, 4 µm; Phenomenex Inc., Torrance, CA, USA) heated to 40°C. A sample volume of 11 µL was injected at a flow rate of 20 µL/min.  Lipids were separated using mobile phases A (100 % H2O, 0.1 % acetic acid) and B (100 % Met-OH, 0.1 % acetic acid) with a gradient starting at 60% B for 0.5 min, steadily increasing to 80% B by 5 min, reaching 95% B by 9 min, holding for 1 min, and then decreasing to 60% B by 12 min. Mass spectrometry analysis was performed on a SCIEX TripleTOF 6600 system using the MRMHR strategy consisting of a TOF-MS experiment looped with multiple MS/MS experiments.  Mass spectrometry spectra were acquired in high-resolution mode (>30,000) using a 100-ms accumulation time per spectrum. Full-scan MS/MS was acquired in high sensitivity mode, with an accumulation time optimized per cycle. Collision energy was set using rolling collision energy with a spread of 15V.  The identity of a component was confirmed using PeakView software (SCIEX). To determine the abundance of the other lipid classes (i.e. acylcarnitines, storage and structural lipids) a direct infusion shotgun lipidomic (MS/MS^ALL^) approach was used as previously described^2^. Briefly, a cocktail of deuterium-labelled and odd chain phospholipid standards from diverse lipid classes was added to 25 µL of plasma. Standards were chosen so that they represented each lipid class and were at designated concentrations chosen to provide the most accurate quantitation and dynamic range for each lipid species. 4 mL chloroform:methanol (1:1, v/v) was added to each sample and the lipid extraction were performed as previously described (Han 2005, Gao 2016). Lipid extraction was automated using a customized sequence on a Hamilton Robotics STARlet system (Hamilton, Reno, NV). Lipid extracts were dried under nitrogen and reconstituted in 68 µl chloroform:methanol (1:1, v/v). Samples were flushed with nitrogen and stored at -20 °C.  Samples were diluted 50 times in isopropanol:methanol:acetonitrile:water (3:3:3:1, by volume) with 2 mM ammonium acetate in order to optimize ionization efficiency in positive and negative modes. Electrospray ionization-MS was performed on a TripleTOF 5600^+^ (SCIEX, Framingham, MA), coupled to a customized direct injection loop on an Ekspert microLC200 system (SCIEX). 50 µL of sample was injected at a flow-rate of 6 µL/min. Lipids were analysed using a customized data independent analysis strategy on the TripleTOF 5600^+^ allowing for MS/MS^ALL^ high resolution and high mass accuracy analysis as previously described^2^.

Lipid levels were determined measuring spectra peak areas relative to internal standards using an in-house library on MultiQuant software (SCIEX). Validation studies – i.e. extraction efficiency, carry over, inter-day coefficient of variation (CV), reproducibility, isotopic correction (type 1 and type 2), and linearity experiments – were all performed.

1. Powell, W. S. Extraction of Eicosanoids from Biological Fluids, Cells, and Tissues. in *Eicosanoid Protocols* 11–24 (Humana Press, 1999). doi:10.1385/1-59259-263-5:11

2. Simons, B. *et al.* Shotgun Lipidomics by Sequential Precursor Ion Fragmentation on a Hybrid Quadrupole Time-of-Flight Mass Spectrometer. *Metabolites* **2,** 195–213 (2012).

**SUPPLEMENTARY TABLES AND FIGURES**

|  |  | | **SPMS** | | | |
| --- | --- | --- | --- | --- | --- | --- |
|  | **SPMS** | | **SPMS-NP** | | **SPMS-P** | |
|  | **Baseline** | **1-Y FU** | **Baseline** | **1-Y FU** | **Baseline** | **1-Y FU** |
| **EDSS median^1^** | 3.5  (3/6.5) | 3.5  (2/6.5) | 3.75  (3/6) | 3.5  (2.5/6) | 3.5  (3/6.5) | 3.5  (2/6.5) |
| **25-FWT, seconds^1^** | 8.7  (5/18.6) | 8.5  (4.3/20) | 7.5  (5/13.8) | 6.7  (4.3/12.4) | 10.7  (6.5/18.6) | 11.5  (6.2/20) |
| **Change in T2 lesion volume, mL^2^** | N/A | -0.02  (-1.7/+1.0) | N/A | -0.51  (-1.7/+1.0) | N/A | +0.36  (+0.1/+1.0) |

**Supplementary Table S1. Clinical and T2 lesion volume changes at baseline and follow-up in SPMS patients.**

Abbreviations: SPMS = secondary progressive MS; SPMS-NP = secondary progressive MS with non-progressed disability; SPMS-P = secondary progressive MS with progressed disability. Unless specified all values are express as mean (range). SPMS, n = 11; SPMS-NP, n = 6; SPMS-P, n = 5.

^1^ Wilcoxon signed rank test was used to detect differences between Baseline and 1-Y FU.

^2^Mann-Whitney U test was applied to compare SPMS-NP vs SPMS-P.

No statistical differences between groups were detected.

|  |  | | **RRMS** | | | |
| --- | --- | --- | --- | --- | --- | --- |
|  | **RRMS** | | **RRMS-S** | | **RRMS-P** | |
|  | **Baseline** | **1-Y FU** | **Baseline** | **1-Y FU** | **Baseline** | **1-Y FU** |
| **EDSS** | 1.0  (0-3.5) | 1.0  (0-3) | 1.0  (0-3.5) | 1.0  (0-2) | 1.0  (0-2) | **1.5***  **(0-3)** |

**Supplementary Table S2. EDSS score at baseline and follow-up in RRMS patients.**

RRMS = relapsing-remitting MS; RRMS-S = relapsing-remitting MS with stable disability; RRMS-P = relapsing-remitting MS with progressed disability. Values are express as median (range). RRMS, n = 24; RRMS-S, n = 13; RRMS-P, n = 11.

*p<0.05, Wilcoxon signed rank test for paired samples Baseline vs 1-Y FU.


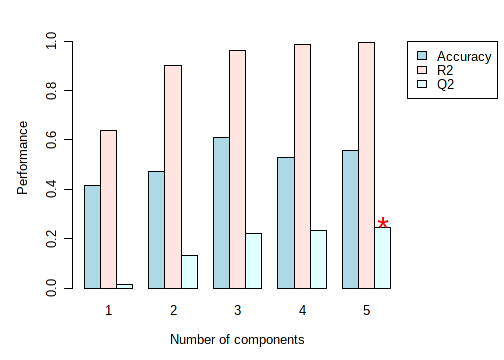


**Supplementary Figure S1. PLS-DA model performance.** Graph shows the performance overview with five components of the PLS-DA model for membrane lipids.
